# When Microneedling Backfires: A Hidden Risk of Hair Shaft Fragility in AGA Patients

**DOI:** 10.1111/jocd.70682

**Published:** 2026-01-19

**Authors:** Wen Xu, Jinlong luo, Yeqin Dai

**Affiliations:** ^1^ Department of Dermatology Hangzhou Third People's Hospital, Hangzhou Third Hospital Affiliated to Zhejiang Chinese Medical University Hangzhou China; ^2^ Department of Dermatology Zhuji Third People's Hospital Zhuji China

**Keywords:** androgenetic alopecia, hair shaft fragility, iatrogenic damage, microneedling therapy, trichoscopy

To the Editor,

1

Microneedling is increasingly used as an adjunctive treatment for androgenetic alopecia (AGA), aiming to enhance transdermal delivery of minoxidil and activate perifollicular signaling through controlled dermal injury [1, 2]. While short‐term efficacy has been reported, the safety margins of low‐depth yet high‐frequency protocols remain incompletely defined. Notably, hair‐shaft structural damage has not been systematically described as a complication of microneedling for AGA. Here, we report two patients who developed paradoxical cosmetic deterioration characterized by hair‐shaft fragility and breakage following repeated low‐depth microneedling, despite an initial favorable response.

Patient 1 was a 37‐year‐old man with a 3‐year history of AGA who underwent weekly microneedling using a 500‐μm roller, combined with twice‐daily topical 5% minoxidil and oral finasteride (1 mg daily) for 12 months. No topical anesthesia or occlusive dressing was used. Clinical improvement during the first 9 months was reported by the patient and supported by serial clinical photography and trichoscopy, whereas diffuse thinning developed over the final 3 months. Trichoscopy at the time of deterioration revealed anisotrichosis and numerous short, broken hairs consistent with shaft fragility (Figure 1a,b). Concurrent scanning electron microscopy (SEM) demonstrated focal cuticle–cortex delamination, widened inter‐scale spaces, and deformation of cuticular plates, consistent with mechanical hair‐shaft trauma (Figure 1c). The patient denied medication changes, systemic illness, or altered grooming practices. Scalp examination showed no erythema, folliculitis, or scale.

**FIGURE 1:**
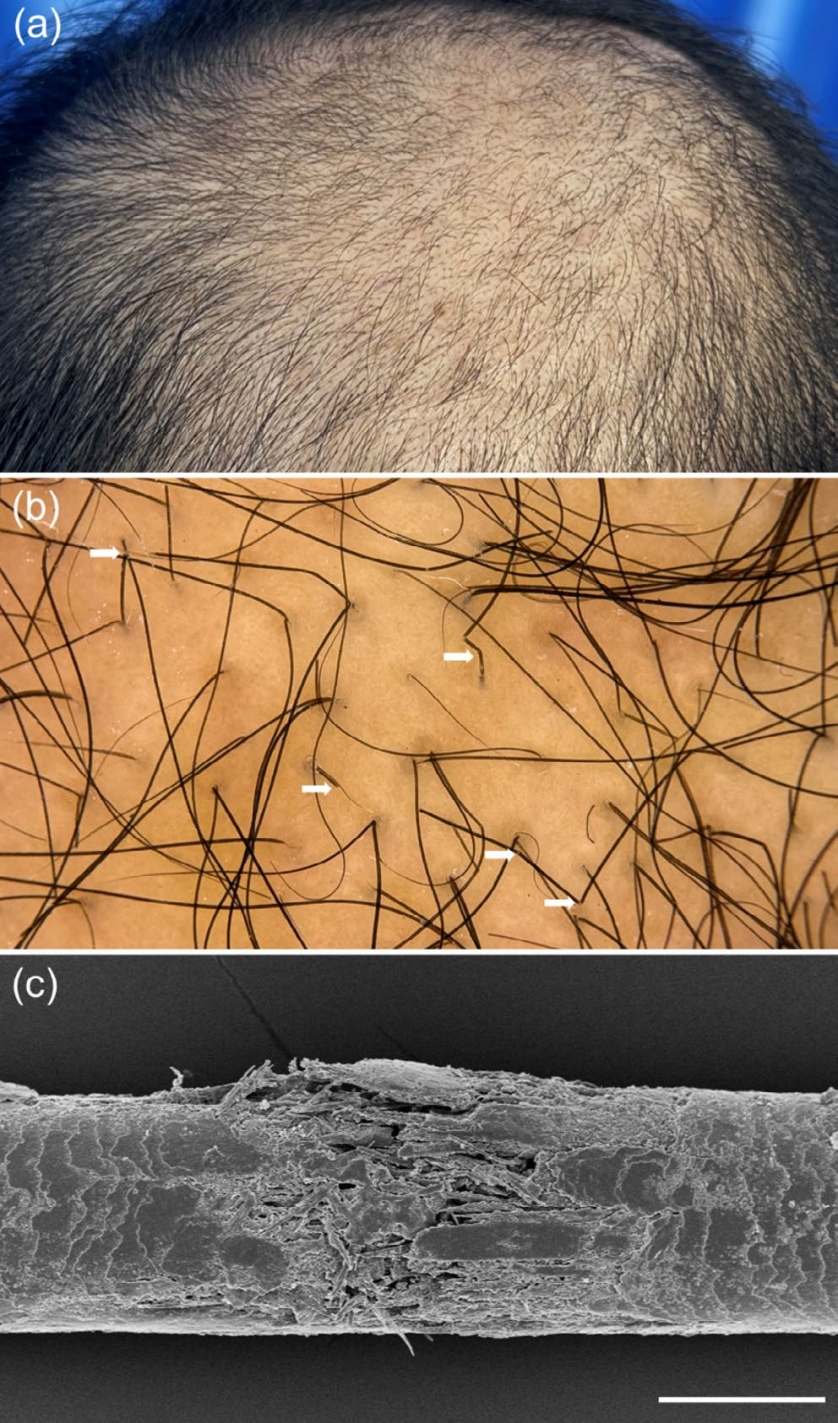
Hair‐shaft fragility after microneedling in a 37‐year‐old male with AGA. (a) Clinical vertex view demonstrating marked worsening of vertex alopecia. (b) Trichoscopic image demonstrating multiple broken hairs (white arrows). (c) Scanning electron microscopy image showing structural disruption of a hair shaft; scale bar = 50 μm.

Patient 2 was a 45‐year‐old woman with a 10‐year history of progressive vertex thinning who received the same weekly microneedling protocol with topical 5% minoxidil. Hair appearance improved during the first 3 months, based on patient report and clinical inspection, but deteriorated by month five, with prominent short, broken hairs and a scissor‐cut pattern at the vertex (Figure 2a,b). SEM revealed lifted, fragmented, and detached cuticular scales, consistent with mechanical shaft injury (Figure 2c). She reported no recent physical trauma, new hair products, or changes in hair‐care routines. Scalp examination was otherwise unremarkable.

**FIGURE 2:**
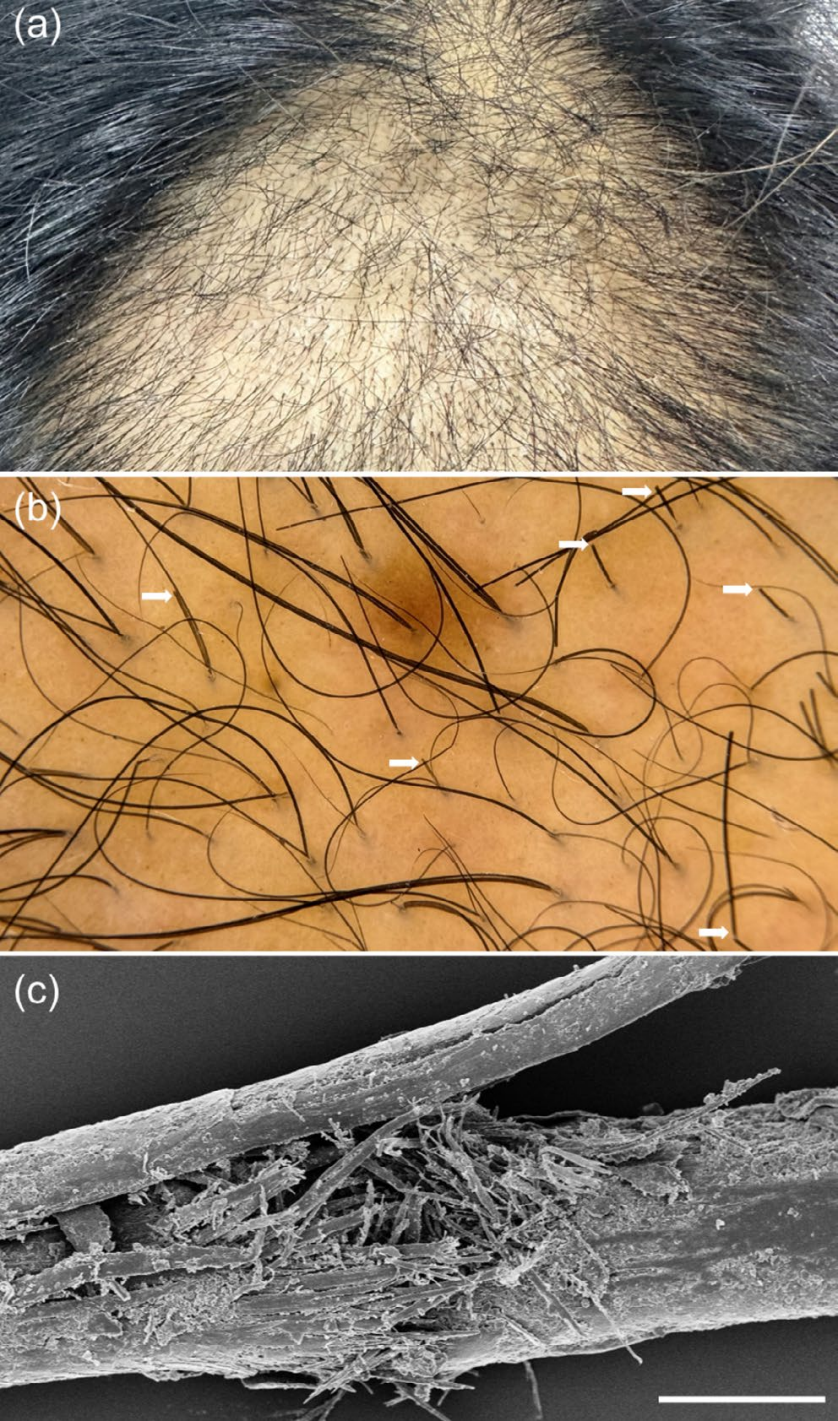
Hair‐shaft fragility after microneedling in a 45‐year‐old female with AGA. (a) Clinical vertex view demonstrating marked worsening of vertex alopecia. (b) Trichoscopic image demonstrating multiple broken hairs (white arrows). (c) Scanning electron microscopy image showing structural disruption of a broken hair shaft; scale bar = 50 μm.

Both cases followed a biphasic course: initial cosmetic improvement followed by reduced apparent hair bulk attributable to fracture‐shortened fibers rather than increased shedding. This pattern argues against telogen effluvium, which typically presents with intact fibers and diffuse shedding [3]. The convergence of trichoscopic findings (broken hairs) and SEM‐confirmed cuticular disruption supports iatrogenic hair‐shaft damage as the proximate process [4]. Trichotillomania, alopecia areata, traction alopecia, and chemical injury were unlikely based on clinical history and trichoscopy. The absence of erythema, pustules, or perifollicular scale further argued against an active inflammatory scalp disorder [5, 6].

Even at a depth of 500 μm, repetitive weekly microneedling may impose cumulative micro‐shear and torsional stress on emerging hair fibers. Procedural variables—including number of passes, direction relative to hair growth, pressure applied, device architecture, and operator technique—may influence the degree of mechanical stress and were not standardized in routine practice. Roller‐based devices can generate angled puncture tracks and transient fiber engagement near follicular exit sites, whereas pen‐type devices distribute force differently. Concurrent minoxidil use may alter fiber hydration and surface friction, potentially lowering the fracture threshold of miniaturized shafts. Insufficient recovery intervals may further impair cuticle re‐compaction, particularly at convex scalp regions such as the vertex. These mechanisms remain hypothesis‐generating, but are supported by the observed SEM patterns of cuticular lifting and plate detachment.

Incorporation of microneedling into AGA management should include monitoring hair‐fiber integrity in addition to density. Routine trichoscopy may allow early detection of shaft fragility and inform protocol adjustment. Extending treatment intervals, standardizing procedural parameters, stroking in the direction of hair growth, and adopting gentle post‐procedure care may reduce the risk of shaft injury while preserving therapeutic benefit.

## Funding

This work was supported by the Hangzhou Joint Fund of the Zhejiang Provincial Natural Science Foundation of China (Project No. LHZSZ24H110001) and the Construction Fund of Key Medical Disciplines of Hangzhou (Dermatology and Venereology, No. 2025HZGF07).

## Ethics Statement

Approved by the Research Ethics Committee of Hangzhou Third People's Hospital; approval #2022KA058.

## Consent

The patients in this manuscript have given written informed consent to the publication of their case details.

## Conflicts of Interest

The authors declare no conflicts of interest.

## Data Availability

The data that support the findings of this study are available from the corresponding author upon reasonable request.
